# Photosynthetic response of *Chlamydomonas reinhardtii* and *Chlamydomonas* sp. 1710 to zinc toxicity

**DOI:** 10.3389/fmicb.2024.1383360

**Published:** 2024-04-08

**Authors:** Di Zhan, Yue Liu, Na Yu, Chunbo Hao

**Affiliations:** ^1^Center for Geomicrobiology and Biogeochemistry Research, State Key Laboratory of Biogeology and Environmental Geology, China University of Geosciences, Beijing, China; ^2^School of Earth Sciences and Resources, China University of Geosciences, Beijing, China; ^3^School of Water Resources and Environment, China University of Geosciences, Beijing, China

**Keywords:** zinc, *Chlamydomonas*, chlorophyll fluorescence parameters, non-photochemical quenching, rapid light curve, fast chlorophyll fluorescence induction curve

## Abstract

Zinc (Zn) is an essential trace element but can lead to water contamination and ecological deterioration when present in excessive amounts. Therefore, investigating the photosynthetic response of microalgae to Zn stress is of great significance. In this study, we assessed the photosynthetic responses of neutrophilic *Chlamydomonas reinhardtii* and acidophilic *Chlamydomonas* sp. 1710 to Zn exposure for 96 h. The specific growth rate (μ), chlorophyll-a (Chl-a) content, and chlorophyll fluorescence parameters were determined. The results demonstrated that *Chlamydomonas* sp. 1710 was much more tolerant to Zn than *C. reinhardtii*, with the half-maximal inhibitory concentration (IC50) values of 225.4 mg/L and 23.4  mg/L, respectively. The μ and Chl-a content of *C. reinhardtii* decreased in the presence of 15  mg/L Zn, whereas those of *Chlamydomonas* sp. 1710 were unaffected by as high as 100  mg/L Zn. Chlorophyll fluorescence parameters indicated that the regulation of energy dissipation, including non-photochemical quenching, played a crucial role in Zn stress resistance for both *Chlamydomonas* strains. However, in the case of *C. reinhardtii*, non-photochemical quenching was inhibited by 5  mg/L Zn in the first 48 h, whereas for *Chlamydomonas* sp. 1710, it remained unaffected under 100  mg/L Zn. *Chlamydomonas* sp. 1710 also exhibited a 20 times stronger capacity for regulating the electron transfer rate than *C. reinhardtii* under Zn stress. The light energy utilization efficiency (α) of *Chlamydomonas* sp. 1710 had the most highly non-linear correlation with μ, indicating the energy utilization and regulation process of *Chlamydomonas* sp. 1710 was well protected under Zn stress. Collectively, our findings demonstrate that the photosystem of *Chlamydomonas* sp. 1710 is much more resilient and tolerant than that of *C. reinhardtii* under Zn stress.

## Highlights

- *Chlamydomonas* sp. 1710 is more Zn tolerant than *Chlamydomonas reinhardtii.*
- Non-photochemical quenching of *C. reinhardtii* was inhibited by 5 mg/L Zn, whereas that of *Chlamydomonas* sp. 1710 was stable under 100 mg/L.
- The electron transfer regulation of *Chlamydomonas* sp. 1710 was more effective than that of *C. reinhardtii* under Zn stress.
- The light energy utilization and regulation of *Chlamydomonas* sp. 1710 was well protected under Zn stress.

## Introduction

Zinc (Zn) is the 24th most abundant element in the Earth’s crust (Wong and Chau, 1990). As an essential trace element, Zn serves as a cofactor for numerous enzymes in various biological processes of plants, including photosynthesis (Broadley et al., 2007). However, excessive Zn can hinder the ability of plants to absorb other divalent metals, such as copper, iron, and calcium, leading to an imbalance of cellular redox processes, ultimately inhibiting photosynthesis (Broadley et al., 2007). Zn pollution mainly occurs in water bodies, originating primarily from industrial activities such as mining, metallurgy, and metal manufacturing. Acid mine drainage (AMD) is an important contributor to water contamination of Zn (Simate and Ndlovu, 2014; Jiang et al., 2023).

Microalgae are crucial components of aquatic environments, serving as primary producers to provide the basic nutrition for heterotrophic organisms (Rizwan et al., 2018). In recent decades, microalgae have garnered increasing attention due to their promising potential in biofuel, food manufacturing, and environmental industry (Spolaore et al., 2006; Zhu et al., 2022; Thanigaivel et al., 2023). *Chlamydomonas reinhardtii* (*C. reinhardtii*) has been widely used as a model organism for genetic and biochemical studies, with its typical habitat being freshwater environments with neutral to slightly alkaline pH optimum (Rochaix, 1995). However, acidophilic algae are the main photosynthetic organisms in AMD, and they contribute to removal of Zn from AMD through two main mechanisms: (1) accumulation of Zn by direct uptake or cohesion by secreting chelating agents such as organic acids and extracellular polymeric substances; (2) providing carbon sources for heterotrophic microorganisms able to increase the pH of the water body, such as sulfate-reducing bacteria, leading to Zn precipitation (Das et al., 2009; Mathimani et al., 2023). The removal of Zn by algae relies on their strong tolerance to Zn toxicity, and large amounts of biomass must be generated to maximize the bioremediation potential of algae. Therefore, photosynthesis, the most fundamental biological process in algae, should receive more attention.

The light energy absorbed by plants or algae has three main fates: (1) transformation into chemical energy through photosynthesis; (2) dissipation in the form of heat; (3) re-emission as fluorescence (i.e., chlorophyll fluorescence). Chlorophyll fluorescence is an important parameter measured in evaluating the physiological state of plants. The first chlorophyll fluorescence measurements were conducted in the 1960s, and this method achieved rapid development in the 1980s when pulse amplitude modulation technology was proposed (Schreiber et al., 1986). F_0_ (initial fluorescence) and F_m_ (maximum fluorescence) are the two basic fluorescence parameters from which maximal photosystem II (PSII) quantum yield (F_v_/F_m_) is calculated (Kitajima and Butler, 1975; Genty et al., 1989; van Kooten and Snel, 1990). Furthermore, chlorophyll fluorescence parameters have evolved into several mainstream branches, including non-photochemical quenching (NPQ) parameters (Schreiber et al., 1986; Kramer et al., 2004), fast chlorophyll fluorescence induction curve (OJIP) parameters (Strasser et al., 2000), and rapid light curve (RLC) parameters (White and Critchley, 1999). Due to their convenience, reproducibility, sensitivity, and accuracy, chlorophyll fluorescence parameters are widely used to investigate photosynthetic responses to various stressors, such as heavy metals (Gebara et al., 2023), salinity (Guermazi et al., 2023), and heat stress (Liang et al., 2023).

Despite the wide utilization of NPQ, OJIP, and RLC parameters, previous studies have largely focused on only one of the aforementioned approaches (Marečková et al., 2019; Almeida et al., 2021; Gebara et al., 2023). Additionally, the temporal variations of these parameters are rarely accounted for, and few studies have compared the chlorophyll fluorescence characteristics across different algal species. In this study, quantum yield, the efficiency of the oxygen evolution complex (OEC), NPQ parameters, OJIP parameters, and RLC parameters were simultaneously used to evaluate the photosynthetic status of two *Chlamydomonas* strains, *C. reinhardtii* and *Chlamydomonas* sp. 1710, which are Zn-sensitive and Zn-tolerant, respectively. Additionally, we evaluated the growth and chlorophyll-a (Chl-a) content of the two *Chlamydomonas* strains. Principal components analysis (PCA), Pearson correlation, and linear/non-linear regression models were used to analyze the relationship between specific growth rate (μ), Chl-a content, and chlorophyll fluorescence parameters. Finally, the chlorophyll fluorescence parameters that were most suitable as indicators of growth and physiological response were identified for the two *Chlamydomonas* strains. Taken together, our findings provide comprehensive insights into the application of different chlorophyll fluorescence parameters for the evaluation of two different *Chlamydomonas* species under Zn stress.

## Materials and methods

### Algal cultures and Zn exposure

*Chlamydomonas reinhardtii* UTEX 90 was generously provided by the National Aquatic Germplasm Resource Bank of the Institute of Hydrobiology, Chinese Academy of Sciences (Wuhan, Hubei, China). *Chlamydomonas* sp. 1710 was isolated from the collected surface water of an AMD in Anhui Province, China. A photoautotrophic medium (1.89 mM (NH_4_)_2_SO_4_, 2.03 mM MgSO_4_·7H_2_O, 0.09 mM CaCl_2_, 2.20 mM KH_2_PO_4_, 0.51 mM NaCl, 17.91 μM FeSO_4_·7H_2_O, 7.72 μM ZnSO_4_·5H_2_O, 46.26 μM H_3_BO_3_, 9.15 μM MnCl_2_·4H_2_O, 1.61 μM Na_2_MoO_4_·2H_2_O, 0.17 μM Co(NO_3_)_2_·6H_2_O, 0.32 μM CuSO_4_·5H_2_O, 26.86 μM Na_2_EDTA·2H_2_O, 40.93 nM D-biotin, 7.38 nM cobalamin, 296.50 nM thiamine) was used. The pH was set to 3, the same as the original water sample, and the temperature was 25°C. 20 μL water sample was serially diluted (10^−3^–10^−6^) using sterile ddH_2_O (18.20 MΩ·cm, Millipore Corporation, Bedford, MA) and inoculated onto agarose plates for two weeks. A single colony was picked and inoculated again onto plates. This procedure was repeated a few times to obtain pure isolate.

Both strains were cultivated in the photoautotrophic medium mentioned above. The pH of the culture medium was adjusted to their optimum (7.0 for *C. reinhardtii* and 3.0 for *Chlamydomonas* sp. 1710). Algal cultivation was conducted in custom-made glass tubes at 25°C. Prior to inoculation, the culture media were autoclaved for 20 min at 121°C. The growth of *Chlamydomonas* strains was monitored by measuring the optical density at 750 nm (OD_750_). *Chlamydomonas* strains were maintained under white light (100 μE·m^−2^·s^−1^) and continuous air injection to reach an OD_750_ of 0.25, which indicated its logarithmic phase. Next, Zn exposure experiments were conducted in a 250 mL glass conical flask containing 100 mL *Chlamydomonas* culture (OD_750_ = 0.25). The Zn concentration range was 0, 5, 15, 30 mg/L for *C. reinhardtii* and 0, 30, 100, 300, 600 mg/L for *Chlamydomonas* sp. 1710. The cells were exposed to Zn for a total of 96 h under 16:8 h (day:night) cycles of white light (100 μE·m^−2^·s^−1^) at 25°C, without air injection. The culture flasks were gently shaken every 12 h. Before use, all glassware was washed with neutral detergent and maintained in 1 M HNO_3_ overnight. All cultures were prepared in triplicate.

### Growth rate determination

OD_750_ is linearly correlated with algal biomass (Tang et al., 2011). Therefore, this parameter was used to determine the growth of *Chlamydomonas* strains in our study. The specific growth rate (μ) was calculated as follows:


$$\mu_{n-0}=\frac{\ln\;\left(N_{b}\right)-\ln\left(N_{a}\right)}{t_{b}-t_{a}}$$

where μ_n-0_ is the average specific growth rate within the time n, N_b_ is the OD_750_ at time b (t_b_), and N_a_ is the OD_750_ at time a (t_a_).

### Chl-a content determination

Chl-a content was determined via the spectrometric method (Goodwin, 1965) at 24, 48, 72, 96 h. Briefly, a 2 mL sample of *Chlamydomonas* suspension was washed using 0.02 M phosphate buffered saline (pH 7.0), and centrifuged for 3 min at 8000 g and 25°C three times to eliminate any interference caused by acidity and Zn ions. The pellet was then extracted using 2 mL of 95% acetone combined with ultrasonication (75 W, 2 min). The extract was then centrifuged for 10 min at 16,000 g and 4°C. The absorbance of the supernatant was measured at 650 and 750 nm. Chl-a content (C_a_) was calculated as follows:


$$C_{a}=25.5\times \left(A650–A750\right)$$

where A650 and A750 represent the absorbance of supernatant at 650 nm and 750 nm, respectively.

### Chlorophyll fluorescence measurements

The chlorophyll fluorescence parameters of *Chlamydomonas* sp. 1710 and *C. reinhardtii* exposed to Zn at 24, 48, 72 and 96 h were measured using an AquaPen-C 100 fluorometer (Photon Systems Instruments, Czech Republic) (Appendix 1). Two milliliters samples of *Chlamydomonas* cultures were kept in the dark at 25°C for at least 15 min to ensure that all PSII reaction centers were in an oxidized state. Prior to each measurement, the *Chlamydomonas* cultures were homogenized via gentle inversion.

To assess maximal PSII quantum yield (F_v_/F_m_) and the efficiency of the oxygen evolution complex (OEC), the samples were irradiated with a saturating light pulse (1,500 μmol·m^−2^·s^−1^) and the fluorescence intensity was recorded. The fluorescence intensity used to calculate effective PSII quantum yield [Y(II)] was obtained from NPQ test described below. To quantify NPQ parameters, the samples were exposed to continuous actinic light (100 μmol·m^−2^·s^−1^) and were irradiated 5 times with saturating light pulses (1,500 μmol·m^−2^·s^−1^) every 12 s. Afterwards, the actinic light was extinguished and the samples were exposed to three saturating light pulses (1,500 μmol·m^−2^·s^−1^) every 26 s.

For the RLC test, the samples were exposed to a series of actinic light intensities (i.e., 10, 20, 50, 100, 300, 500, 1,000 μmol·m^−2^·s^−1^), with each exposure lasting for 60 s. The samples were then irradiated with a saturating light pulse (1,500 μmol·m^−2^·s^−1^) at intervals determined by the shift in actinic light intensity, after which the relative photosynthetic electron transfer rate (ETR) under various light intensities of continuous illumination were obtained.

For the OJIP test, the cells were irradiated with 1,500 μmol·m^−2^·s^−1^ of blue light (450 nm) for 2 s to obtain a curve encompassing four typical phases: the start phase at 50 μs; the intermediate phases J and I at 2 ms and 30 ms, respectively; and the maximal phase P (equal to F_m_ at saturating light conditions). Following the above-described protocols, all relevant chlorophyll fluorescence parameters were measured or calculated.

### Statistical analysis

One-way analysis of variance (ANOVA) with multiple comparison tests was used to identify statistically significant differences among the data under different Zn concentrations. A *p*-value < 0.05 was considered statistically significant. The statistical analyses were conducted using the GraphPad Prism 7.0 software (GraphPad, San Diego, USA).

The acquired data were processed via principal component analysis (PCA) using the OriginPro 2022 software (OriginLab Corporation, Northampton, MA) to resolve the collinearity of complex chlorophyll fluorescence-associated parameters (Wold et al., 1987), including μ, Chl-a content, maximal PSII quantum yield (F_v_/F_m_), efficiency of the oxygen-evolving complex (OEC), effective PSII quantum yield [Y(II)], coefficient of photochemical quenching (qP), non-photochemical quenching (NPQ), coefficient of photochemical quenching (qL), quantum yield of regulated energy dissipation [Y(NPQ)], quantum yield of nonregulated energy dissipation [Y(NO)], coefficient of non-photochemical quenching (qN), relative photochemical quenching [qP(rel)], relative non-photochemical quenching [qN(rel)], relative unquenched fluorescence [UQF(rel)], absorption flux per reaction center (ABS/RC), trapped energy flux per reaction center (TRo/RC), dissipated energy flux per reaction center (DIo/RC), electron transport flux per reaction center (ETo/RC), electron flux reducing end electron acceptors at the PSI acceptor side per reaction center (REo/RC), performance index for energy conservation from photons absorbed by PSII to the reduction of intersystem electron acceptors (PI_ABS), performance index for energy conservation from photons absorbed by PSII to the reduction of PSI and acceptors (PI_total), light energy utilization efficiency (α), and maximal relative photosynthetic electron transport rate (ETR_max_). The obtained principal components (PCs) were used to resolve statistical models, in which the scores represent the distance from the PCs’ origin to every data point, and the loadings represent the contributions of each parameter to each PC (Bro and Smilde, 2014).

Pearson correlation analysis was conducted to identify pairwise correlations between μ, Chl-a content, and all chlorophyll fluorescence parameters using the GraphPad Prism 7.0 software (GraphPad, San Diego, USA). Linear and non-linear regression analyses between μ, Chl-a content, and selected chlorophyll fluorescence parameters were determined with a variable slope model, using the least squares fitting method in the GraphPad Prism 7.0 software (GraphPad, San Diego, USA).

## Results and discussion

### Growth curves and Chl-a content

**Figure 1:**
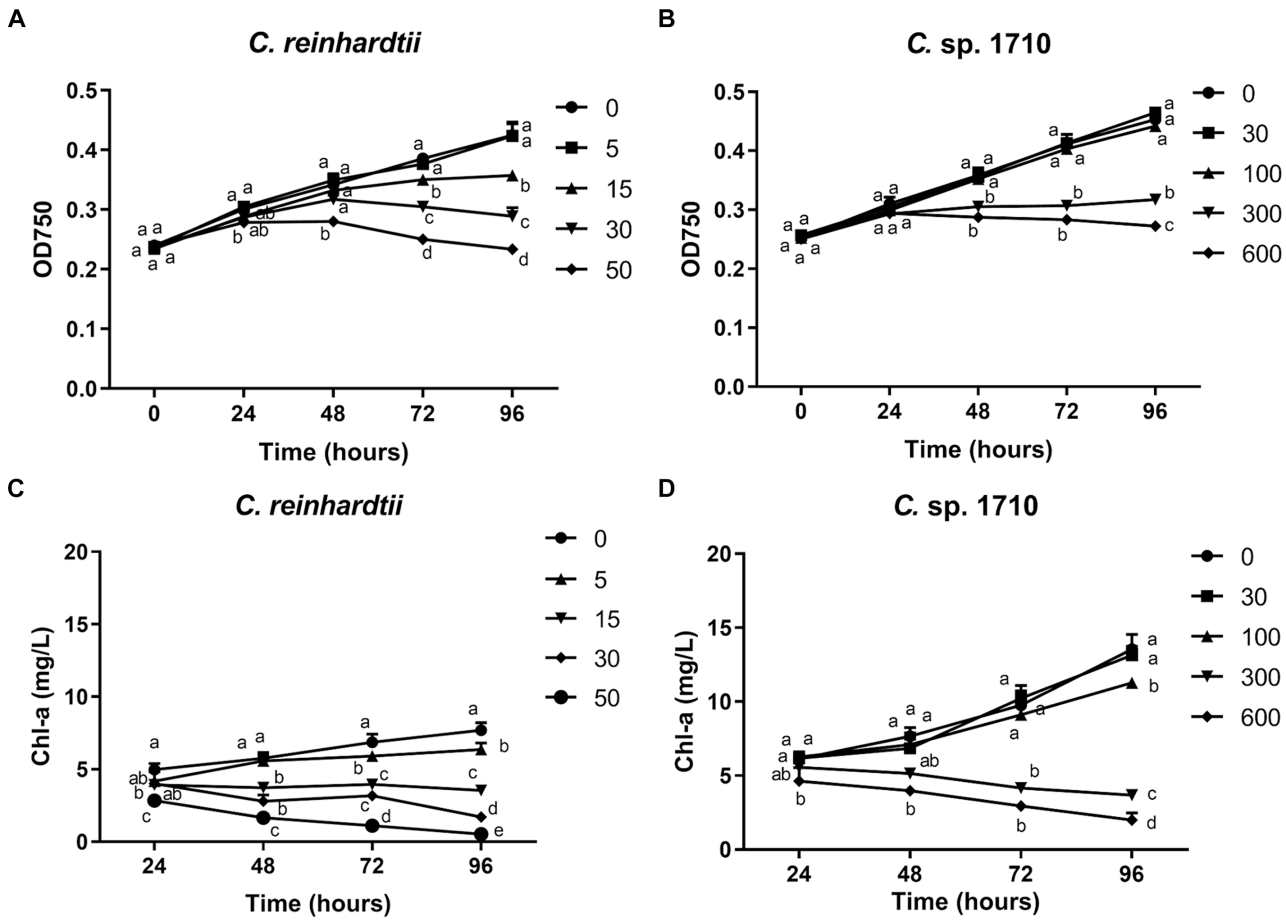
OD_750_ **(A,B)** and Chlorophyll a (Chl-a) content **(C,D)** of *C. reinhardtii* and *Chlamydomonas* sp. 1710 exposed to Zn (mg/L) for 96 h.

The impact of Zn on the growth of *C. reinhardtii* and *Chlamydomonas* sp. 1710 is illustrated in Figures 1A,B. These two species of *Chlamydomonas* exhibited different levels of tolerance toward Zn. 5 mg/L Zn did not exert any side effects on the growth of *C. reinhardtii*, whereas 15 mg/L and 30 mg/L Zn significantly inhibited its growth. In contrast, 30 mg/L and even 100 mg/L Zn did not inhibit the growth of *Chlamydomonas* sp. 1710. The half-maximal inhibitory concentrations (IC50) of *C. reinhardtii* and *Chlamydomonas* sp. 1710 were calculated to be 23.4 mg/L and 225.4 mg/L, respectively. These results aligned with previous comparative studies between acidophilic and neutrophilic algae, indicating *Chlamydomonas* sp. 1710 was more tolerant to Zn stress than *C. reinhardtii* (Abinandan et al., 2019; Mikulic and Beardall, 2021).

Chl-a is a pigment that plays an essential role in algal photosynthesis. The Chl-a content of both *Chlamydomonas* decreased under Zn stress (Figures 1C,D). However, Zn concentration as low as 5 mg/L significantly decreased the Chl-a content of *C. reinhardtii* (Figure 1C). In contrast, for *Chlamydomonas* sp. 1710, 30 and even 100 mg/L Zn did not have a significant inhibitory effect (Figure 1D). The result may be due to the fact that *Chlamydomonas* sp. 1710 had an inherently higher amount of Chl-a than *C. reinhardtii*, with 13.5 mg/L and 7.7 mg/L, respectively, under normal growth at 96 h (Figures 1C,D). This was consistent with previous studies (Pluciński et al., 2023). Furthermore, Zn-induced ROS would cause severe damage to Chl-a within cells (Dong et al., 2023; Pluciński et al., 2023), and *Chlamydomonas* sp. 1710 could have powerful antioxidant enzymes eliminating excessive ROS (del Carmen Romero-Cruz et al., 2024).

### Quantum yield and OEC

Quantum yield is a measure of the number of useful products or events produced per absorbed photon during photosynthesis (Kitajima and Butler, 1975). This parameter represents the energy efficiency from absorbed photons to drive desired chemical or biological reactions. A quantum yield of 1.0 means that every absorbed photon is utilized to produce desired product or event. The maximal PSII quantum yield (F_v_/F_m_) refers to the maximum efficiency of converting light energy into chemical energy through the process of photochemical reactions (Kitajima and Butler, 1975). F_v_/F_m_ typically cannot be reached, and the specific value of parameter is commonly used as an indicator of plant health. Effective PSII quantum yield [Y(II)] represents the authentic quantum conversion rate of algae under normal growth condition (Genty et al., 1989).

**Figure 2:**
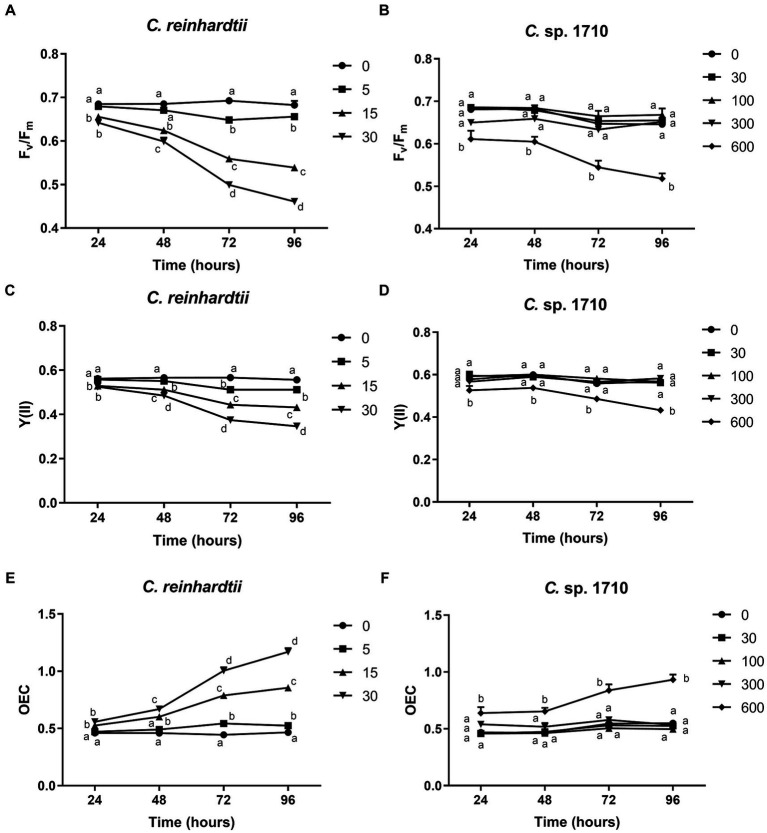
Maximal PSII quantum yield (F_v_/F_m_) **(A,B)**, effective PSII quantum yield [Y(II)] **(C,D)**, and OEC **(E,F)** of *C. reinhardtii* and *Chlamydomonas* sp. 1710 exposed to Zn (mg/L) for 96 h.

The quantum yield of both *C. reinhardtii* and *Chlamydomonas* sp. 1710 responded divergently to Zn exposure (Figures 2A–D). The F_v_/F_m_ and Y(II) values of *C. reinhardtii* correlated negatively with Zn concentration, and their trends aligned with the growth curves presented in Figures 1A,B. Moreover, this negative correlation became more pronounced over time. In 96 h, F_v_/F_m_ decreased from 0.68 at 0 mg/L Zn to 0.46 at 30 mg/L Zn. However, a concentration up to 300 mg/L did not have any inhibitory effect on the F_v_/F_m_ and Y(II) of *Chlamydomonas* sp. 1710. This result indicated that the quantum yield of *C. reinhardtii* was inhibited by Zn, and the inhibition occurred at a concentration as low as 5 mg/L, whereas that of *Chlamydomonas* sp. 1710 could be stable up to 300 mg/L. The high stability of quantum yield to heavy metal stress was also reported in mercury-tolerant algae (Juneau et al., 2001).

The oxygen evolution complex is a protein complex located in the thylakoid membrane of the chloroplast, specifically within the PSII complex (Cady et al., 2008). This complex plays a crucial role in the light-dependent reactions of photosynthesis by facilitating the conversion of water molecules into molecular oxygen, protons, and electrons (Cady et al., 2008). OEC is defined by its ability to facilitate the process of oxygen evolution during photosynthesis (Maxwell and Johnson, 2000). For *C. reinhardtii*, the OEC exhibited a positive correlation with Zn concentration, and increased from 0.47 at 0 mg/L Zn to 1.17 at 30 mg/L Zn (Figure 2E). Conversely, the OEC of *Chlamydomonas* sp. 1710 remained unaffected at <300 mg/L Zn concentration but increased from 0.53 to 0.93 when the Zn concentration increased from 0 to 600 mg/L (Figure 2F). The variation trends of OEC were contrary to those of the quantum yield, which was consistent with their theoretical trends obtained via calculations, as well as the biological implications that the efficiency of light energy conversion was inhibited by a high level of Zn, thereby reinforcing the photo-oxidation of water (Kitajima and Butler, 1975; Kriedemann et al., 1985).

Both the quantum yield and OEC of *C. reinhardtii* and *Chlamydomonas* sp. 1710 exhibited divergent trends. For *C. reinhardtii*, the variations of F_v_/F_m_, Y(II), and OEC were consistent with those of the growth curves and Chl-a contents (Figures 1, 2). However, for *Chlamydomonas* sp. 1710, although algal growth was significantly inhibited by 300 mg/L Zn (Figure 1B), its F_v_/F_m_, Y(II) and OEC remained unaffected. This result suggests that low Zn concentrations can directly impair the photon conversion capacity of *C. reinhardtii*’s photosystem, hence suppressing its growth, whereas that of *Chlamydomonas* sp. 1710 could tolerate up to 300 mg/L Zn. Therefore, the observed variations in chlorophyll fluorescence parameters suggested that, unlike *C. reinhardtii*, *Chlamydomonas* sp. 1710 likely possesses important protective mechanisms for its photosystem, such as antioxidant enzymes and non-photochemical quenching (Pluciński et al., 2023; del Carmen Romero-Cruz et al., 2024).

### NPQ parameters

In the past few decades, several NPQ parameters were proposed by different research groups, including coefficient of photochemical quenching (qP), coefficient of non-photochemical quenching (qN) (Schreiber et al., 1986), relative photochemical quenching [qP(rel)], relative non-photochemical quenching [qN(rel)] (Buschmann, 1995), non-photochemical quenching (NPQ) (Bilger and Björkman, 1990), and coefficient of photochemical quenching (qL) (Kramer et al., 2004). Among these, qP, qP(rel), and qL represent photochemical quenching, whereas qN, qN(rel), and NPQ represent non-photochemical quenching. Photochemical quenching represents the energy transfer that occurs during photosynthesis (Ort and Baker, 2002), whereas non-photochemical quenching is an important regulation mechanism exhibited by plants in response to abiotic stress, involving the heat dissipation of light energy absorbed by light-harvesting antenna (Demmig-Adams et al., 2014). Furthermore, Y(NO) represents the passively dissipated energy in the form of heat and fluorescence, mainly due to closure of PSII reaction centers, which increases during photodamage (Klughammer and Schreiber, 2008). Y(NPQ) reflects the regulated energy dissipation through the non-photochemical quenching process. UQF(rel) represents the relative unquenched fluorescence mainly caused by closed PSII reaction centers (Juneau et al., 2005) and it usually increases with more severe stress (Ranjbarfordoei et al., 2006; Deblois et al., 2013).

**Figure 3:**
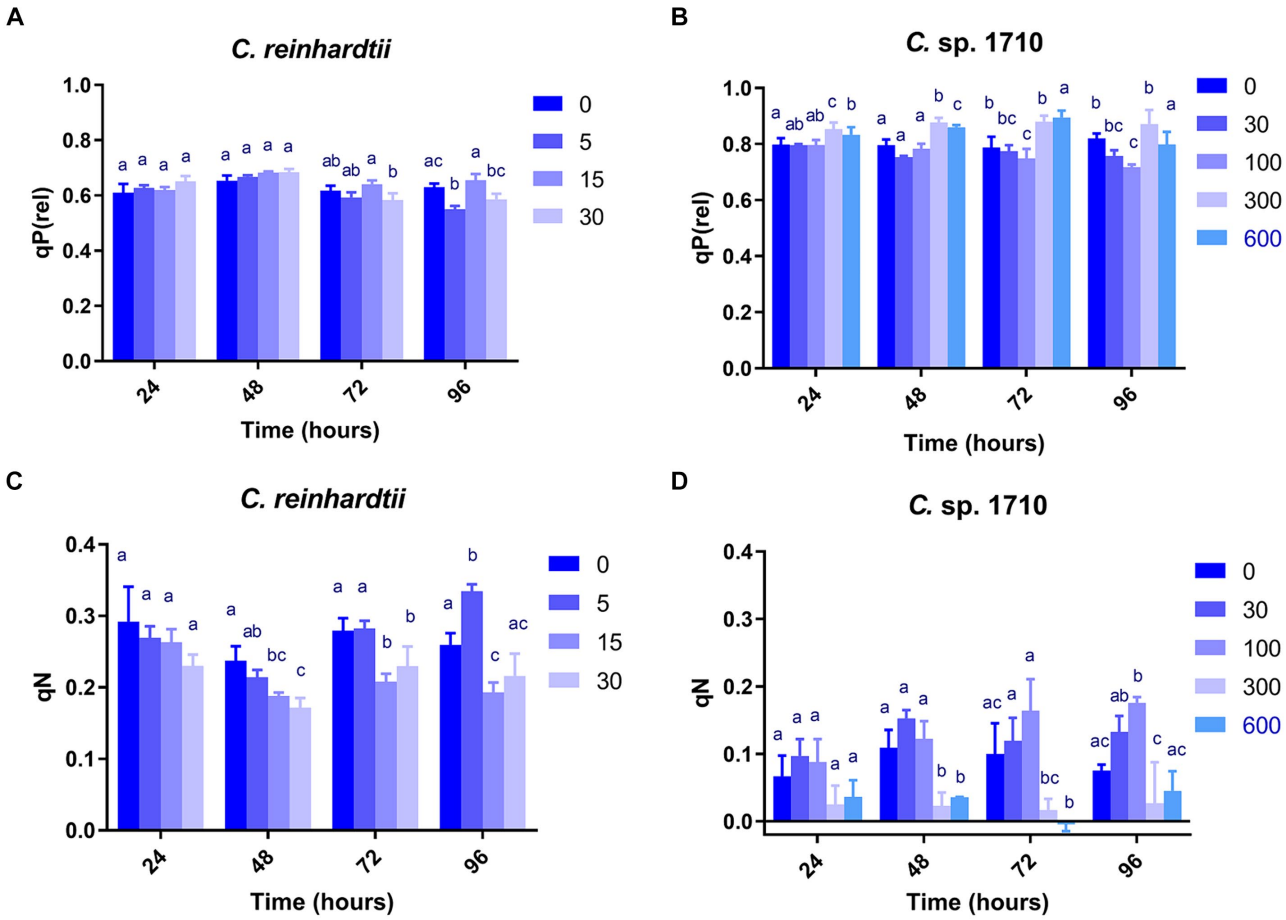
Relative photochemical quenching [qP(rel)] **(A,B)** and coefficient of non-photochemical quenching (qN) **(C,D)** of *C. reinhardtii* and *Chlamydomonas* sp. 1710 exposed to Zn (mg/L) for 96 h.

qP, qP(rel), and qL exhibited very divergent trends for the two *Chlamydomonas* strains (Figures 3A,B; Supplementary Figures S1A–D). qL remained nearly constant across all Zn concentrations and exposure times for both *C. reinhardtii* and *Chlamydomonas* sp. 1710. Therefore, it may not be an appropriate parameter to evaluate the performance of the photosystem II for the two *Chlamydomonas* strains. However, qP(rel) and qP varied with Zn concentration and exposure time. In the first 48 h, the qP(rel) of *C. reinhardtii* increased slightly with increased Zn concentration (Figure 3A), indicating that Zn had no appreciable adverse effect on photosynthesis during this period. Subsequently, the qP(rel) of *C. reinhardtii* decreased with increased Zn concentration, except for 15 mg/L, where it was higher. In contrast, the qP(rel) of *Chlamydomonas* sp. 1710 was unchanged under 30 and 100 mg/L Zn but increased under 300 or 600 mg/L Zn (Figure 3B). The trends of qP were different from that of qP(rel). For *C. reinhardtii*, qP exhibited minimal changes at various Zn concentrations within the first 48 h (Supplementary Figure S1A) but decreased slightly as the Zn concentration increased after 72 h. Conversely, the qP of *Chlamydomonas* sp. 1710 remained unchanged during the initial 72 h even under a Zn concentration as high as 300 mg/L, and only decreased under 600 mg/L Zn at 96 h (Supplementary Figure S1B). This was consistent with previous studies (Baracho et al., 2019; Rocha et al., 2021) reporting that although the Chl-a content of both *C. reinhardtii* and *Chlamydomonas* sp. 1710 significantly decreased upon exposure to Zn, the residual Chl-a still possessed most photosynthetic functions. The results of qP(rel) and qP indicated that, being the most important cellular component of algae, the photosynthetic system would not be impaired primarily by heavy metals due to protective mechanisms, such as enzymatic and non-enzymatic antioxidants, as well as metal transporters (Candido and Lombardi, 2018; Leong and Chang, 2020).

qN, qN(rel), and NPQ exhibited highly similar trends in both *C. reinhardtii* and *Chlamydomonas* sp. 1710 (Figures 3C,D; Supplementary Figures S1E–H). Therefore, the discussion focused on the variations in qN. As a regulatory function, non-photochemical quenching was found to either increase (Nama et al., 2019; Rocha et al., 2021) or decrease (Almeida et al., 2021; Aparicio et al., 2022) with increased concentration of toxic substances. This divergence may be due to the fact that most previous studies have only focused on a single time point without temporal trends. Additionally, previous studies have demonstrated that the mechanisms of non-photochemical quenching are distinct across algae, cyanobacteria, and plants, depending on different proteins and pigments (Giacometti and Morosinotto, 2013; Nowicka, 2020; Lu et al., 2022). In this study, non-photochemical quenching was simultaneously influenced by multiple factors including algae species, Zn concentration, and exposure time. Moreover, the responses of the two species of *Chlamydomonas* occurred at different times. Consistent with previous studies (Dewez et al., 2005), the qN of both *C. reinhardtii* and *Chlamydomonas* sp. 1710 were sensitive to Zn concentration. Under Zn stress, the qN of *C. reinhardtii* decreased from 0.3 to lower than 0.2 (Figure 3C) before 48 h but recovered afterwards. In contrast, under 30 and 100 mg/L, the qN of *Chlamydomonas* sp. 1710 increased from 0.05 to nearly 0.2 (Figure 3D) throughout the entire period. Therefore, non-photochemical quenching appeared to be delayed in *C. reinhardtii* under 5 mg/L Zn, whereas it was stable in *Chlamydomonas* sp. 1710 under 30 and 100 mg/L Zn. On the other hand, the growth of *C. reinhardtii* was unaffected by 5 mg/L Zn, and that of *Chlamydomonas* sp. 1710 also remained unaffected under 30 and 100 mg/L Zn. Consequently, non-photochemical quenching may serve as a crucial protective mechanism for both *C. reinhardtii* and *Chlamydomonas* sp. 1710 under Zn stress, which was consistent with previous studies (Gebara et al., 2023; Pluciński et al., 2023). However, *Chlamydomonas* sp. 1710 had a superior regulation of non-photochemical quenching compared to *C. reinhardtii* and other microalgae (Rocha et al., 2024).

**Figure 4:**
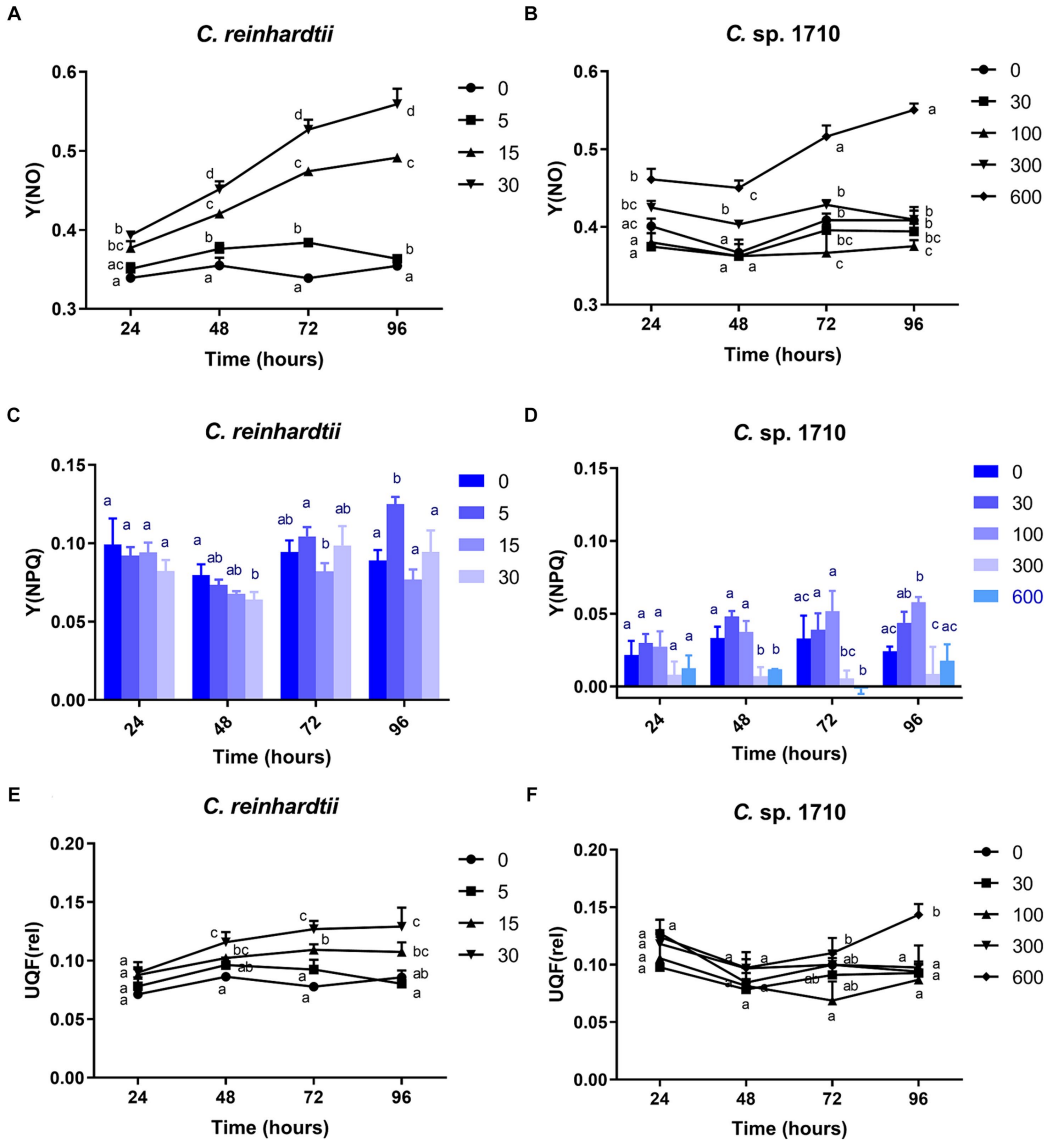
Quantum yield of nonregulated energy dissipation [Y(NO)] **(A,B)**, quantum yield of regulated energy dissipation (Y(NPQ)] **(C,D)**, and relative unquenched fluorescence [UQF(rel)] **(E,F)** of *C. reinhardtii* and *Chlamydomonas* sp. 1710 exposed to Zn (mg/L) for 96 h.

The Y(NO) of *C. reinhardtii* increased with both Zn concentration and exposure time (Figure 4A). The Y(NO) of *C. reinhardtii* was minimally affected at 5 mg/L Zn but increased to >0.5 at 30 mg/L. This indicates that for *C. reinhardtii*, the photosynthetic consumption and non-photochemical quenching were unable to offset the absorbed light energy under severe Zn stress (Sekulska-Nalewajko et al., 2019), resulting in light-induced impairment of the photosystem (Misra et al., 2012). In contrast, 30 and 100 mg/L Zn had negligible effects on the Y(NO) of *Chlamydomonas* sp. 1710, whereas its Y(NPQ) increased significantly (Figures 4B,D). This suggested that *Chlamydomonas* sp. 1710 exhibited robust energy regulation abilities under 30 or 100 mg/L Zn. The UQF(rel) of *C. reinhardtii* had a positive correlation with Zn concentration (Figure 4E), suggesting that Zn inhibited its cellular quenching regulation (Juneau et al., 2005). Conversely, the UQF(rel) of *Chlamydomonas* sp. 1710 was minimally influenced by Zn, indicating an excellent regulatory performance of *Chlamydomonas* sp. 1710 in response to Zn exposure. Interestingly, when combining Y(NO), Y(NPQ), and UQF(rel), *C. reinhardtii* and *Chlamydomonas* sp. 1710 had different photosynthetic responses to Zn, similar to the responses of different kinds of plants to biotic stress (Sekulska-Nalewajko et al., 2019). Under severe Zn stress, the Y(NO) and UQF(rel) of both *C. reinhardtii* and *Chlamydomonas* sp. 1710 increased, whereas Y(NPQ) decreased (Figure 4). However, both strains exhibited resilience to a slight Zn stress, albeit at different response rates. The Y(NO) and UQF(rel) of *C. reinhardtii* increased at 5 mg/L but returned to the baseline level at 96 h (Figures 4A,E). This was attributed to the fact that the Y(NPQ) of *C. reinhardtii* was inhibited before 48 h but began to recover afterwards (Figure 4C). Conversely, the Y(NPQ) of *Chlamydomonas* sp. 1710 immediately increased at 24 h (Figure 4D), whereas Y(NO) and UQF(rel) were maintained (Figures 4B,F). The results of *C. reinhardtii* aligned with previous studies, indicating increased unregulated energy dissipation under abiotic stress (Antunović Dunić et al., 2023; Pluciński et al., 2023). In contrast, *Chlamydomonas* sp. 1710 demonstrated a distinct response, showing superior energy regulation.

### RLC parameters

**Figure 5:**
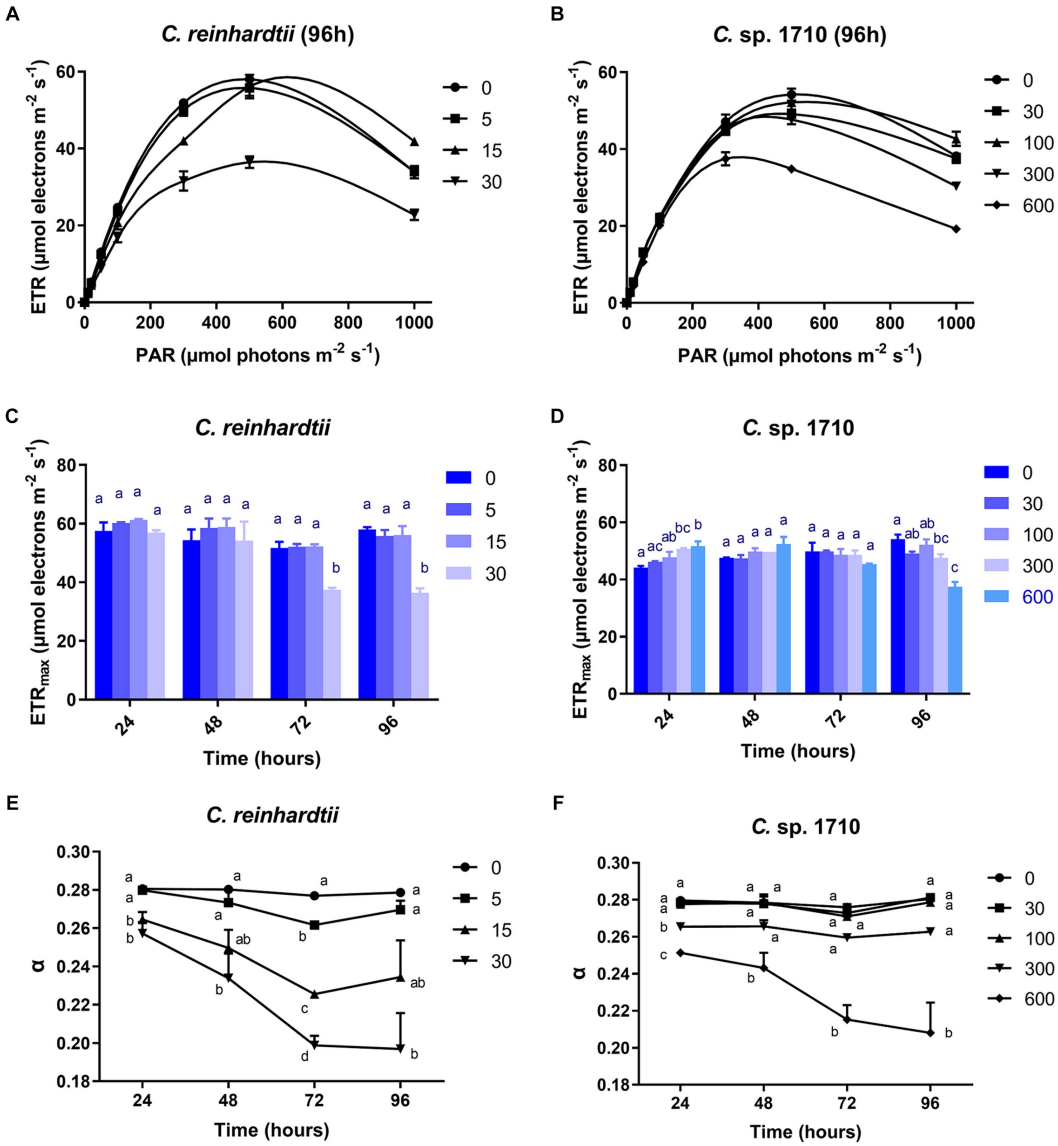
Rapid light curve (RLC) **(A,B)**, maximal relative photosynthetic electron transport rate (ETR_max_) **(C,D)**, and light energy utilization efficiency (α) **(E,F)** of *C. reinhardtii* and *Chlamydomonas* sp. 1710 exposed to Zn (mg/L) for 96 h.

RLC can be used to obtain the electron transport rate (ETR) of the photosystem of green algae under Zn stress, and to further calculate the light energy utilization efficiency (α) (Fu et al., 2012). The ETR of *C. reinhardtii* remained unaffected by Zn within the initial 48 h, but was inhibited significantly by 30 mg/L Zn after 72 h (Figure 5A; Supplementary Figures S2A,C,E). In contrast, for *Chlamydomonas* sp. 1710, the ETR was not inhibited until 96 h by 300 mg/L Zn (Figure 5B; Supplementary Figures S2B,D,F). The varying response patterns of the two *Chlamydomonas* strains did not align with their growth curves (Figures 1A,B). Their heavy metal sensitivity patterns were different from that of *Ankistrodesmus densus*, another green alga (Rocha et al., 2021). However, there were intriguing changes in the trend of ETR_max_, with previous studies demonstrating that ETR_max_ has a good correlation with the growth and pigment content of algae (Bischof et al., 2000; Lüder et al., 2001). The ETR_max_ of *C. reinhardtii* decreased from 58 to 36.5 μmol electrons m^−2^·s^−1^ when Zn concentration increased from 0 to 30 mg/L Zn at 96 h, but remained unchanged under 15 mg/L despite experiencing remarkable growth inhibition under this Zn level (Figure 5C). In contrast, the ETR_max_ of *Chlamydomonas* sp. 1710 was almost unaffected (Figure 5D). The fact that the cells maintained such a high electron transfer rate despite suppressed growth was rather intriguing. Here, we propose two potential explanations: (1) the electron transfer process was effectively protected by cellular regulation mechanisms including non-photochemical quenching or detoxification mechanisms (e.g., enzymatic and non-enzymatic removal of ROS impairing electron transfer) (Hasan et al., 2015; Rezayian et al., 2019); and (2) the high rate of electron transfer served as a protective mechanism (e.g., the enhancement of cyclic electron transfer protected the photosystem by promoting ATP synthesis) (Kalra et al., 2020). Therefore, the results indicated that both *Chlamydomonas* strains responded to Zn stress by maintaining a high electron transfer rate. However, the response of *Chlamydomonas* sp. 1710 was clearly superior given that its ETR_max_ was fairly high under 300 mg/L Zn, whereas that of *C. reinhardtii* was significantly inhibited by even 15 mg/L Zn. Notably, the trends of ETR_max_ were consistent with the growth curve for both *C. reinhardtii* and *Chlamydomonas* sp. 1710, indicating that under Zn stress, the growth of *Chlamydomonas* could be highly influenced by the electron transfer rate, and ETR_max_ could serve as a good growth rate indicator. The transition of ETR_max_ trend at approximately 48 h was also demonstrated by a previous transcriptomic and metabolomic study of *Chlamydomonas* under heavy metal exposure (Jamers et al., 2013).

The light energy utilization efficiency (α) of *C. reinhardtii* was almost unaffected by 5 mg/L Zn, but decreased from 0.28 to 0.2 when Zn concentration increased from 0 to 30 mg/L (Figure 5E). In contrast, the α of *Chlamydomonas* sp. 1710 remained unchanged under 30 and 100 mg/L Zn (Figure 5F). The result indicated that compared to *C. reinhardtii*, *Chlamydomonas* sp. 1710 was more effective at regulating its light energy utilization. Notably, the trend of α was more consistent with that of the growth curve than any other examined parameters, indicating it had the potential to be the best indicative parameter of cell growth. This was in agreement with previous findings in *Chlorella*, another green alga species (Li et al., 2016).

### OJIP parameters

The OJIP test provides insights into the electron flux among the different components of PSII by determining the transient fluorescence of several extremely short time points. Several parameters were derived in this study, including absorption flux per reaction center (ABS/RC), trapped energy flux per reaction center (TRo/RC), dissipated energy flux per reaction center (DIo/RC), electron transport flux per reaction center (ETo/RC), electron flux reducing end electron acceptors at the PSI acceptor side per reaction center (REo/RC), performance index for energy conservation from photons absorbed by PSII to the reduction of intersystem electron acceptors (PI_ABS) (Strasser et al., 2000, 2010).

**Figure 6:**
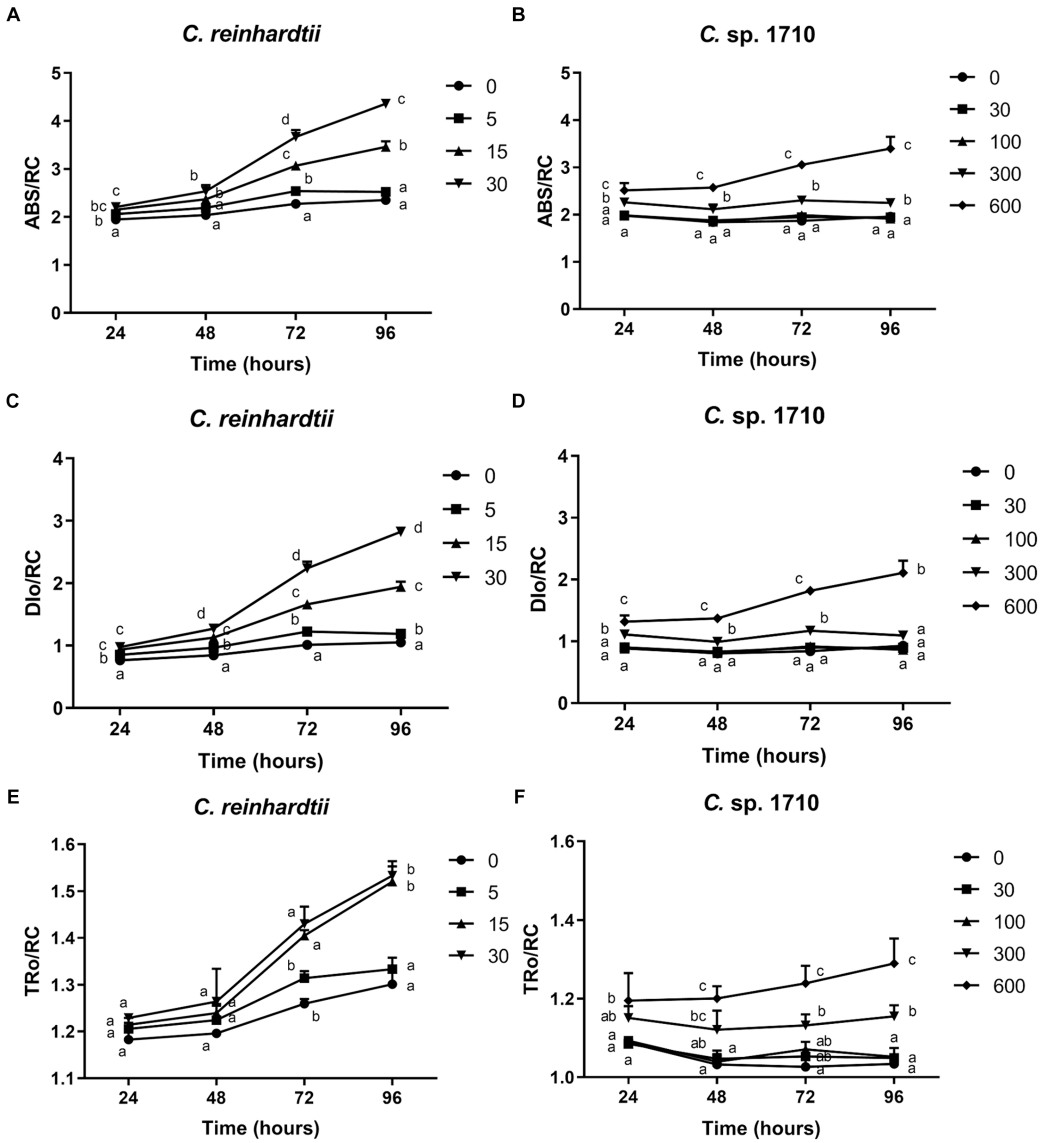
Absorption flux per reaction center (ABS/RC) **(A,B)**, trapped energy flux per reaction center (TRo/RC) **(C,D)**, and dissipated energy flux per reaction center (DIo/RC) **(E,F)** of *C. reinhardtii* and *Chlamydomonas* sp. 1710 exposed to Zn (mg/L) for 96 h.

ABS/RC is the average light energy absorbed by the PSII reaction center, and represents the effective antenna size of an active reaction center (Gomes et al., 2012). The increase in the size of light-harvesting antennae is an induced response under stress conditions that enables plants to compete for light energy (Negi et al., 2020). However, for algal populations, truncation of light-harvesting antennae is considered a very effective pathway for substantially enhancing photosynthetic efficiency and biomass yield (Negi et al., 2020; Kumar et al., 2021). The ABS/RC of *C. reinhardtii* had a positive correlation with Zn concentration, and increased from 2.35 to 4.36 when Zn concentration increased from 0 to 30 mg/L (Figure 6A), suggesting that Zn triggered an increase in the light energy absorbed per reaction center. In contrast, the ABS/RC of *Chlamydomonas* sp. 1710 maintained a fairly constant value under lower than 100 mg/L, but increased from 1.92 to 3.34 when Zn concentration increased from 0 to 600 mg/L (Figure 6B). This increase in the ABS/RC could be attributed to the increase in light-harvesting antennae or the reduction of cell concentration, thus decreasing the competition for light energy (Negi et al., 2020). Light saturation of the electron-transport system inevitably leads to the non-productive dissipation of excessive captured energy (Negi et al., 2020). Therefore, the DIo/RC of *C. reinhardtii* increased from 1.05 to 2.83 when Zn concentration increased from 0 to 30 mg/L, indicating a substantial dissipation of energy when exposed to Zn (Figure 6C). In contrast, The DIo/RC of *Chlamydomonas* sp. 1710 remained stable under lower than 300 mg/L, and increased from 0.87 to 2.11 when Zn concentration increased from 0 to 600 mg/L (Figure 6D). The TRo/RC of *C. reinhardtii* also increased with increased Zn concentration (Figure 6E), albeit to a lesser extent than the increase observed in ABS/RC and DIo/RC, owing to an enhanced passive energy acquisition (Li et al., 2018; Singh et al., 2022). Strikingly, although 5 mg/L slightly increased the ABS/RC and DIo/RC of *C. reinhardtii*, the TRo/RC remained largely unchanged (Figure 6E). This was consistent with previous studies (Liu et al., 2021; Antunović Dunić et al., 2023), indicating that the energy dissipation process offsets excess energy and effectively protected the photosystem complex. However, the TRo/RC of *C. reinhardtii* increased from 1.3 to 1.53 when Zn concentration increased from 0 to 30 mg/L (Figure 6E), showing that the energy dissipation was insufficient. Similar to DIo/RC, the TRo/RC of *Chlamydomonas* sp. 1710 remained unchanged at 30 and 100 mg/L throughout the entire 96 h, and increased from 1.03 to 1.29 when Zn concentration increased from 0 to 600 mg/L (Figure 6F). These results were highly consistent with the NPQ parameters, which demonstrated that energy dissipation, including non-photochemical quenching, were important for the energy regulation in the PSII of the two *Chlamydomonas*. However, the regulation of *Chlamydomonas* sp. 1710 was clearly more effective given that its trapped energy remained constant at Zn concentrations of up to 100 mg/L, whereas *C. reinhardtii* could only tolerate Zn concentrations of 5 mg/L or less.

**Figure 7:**
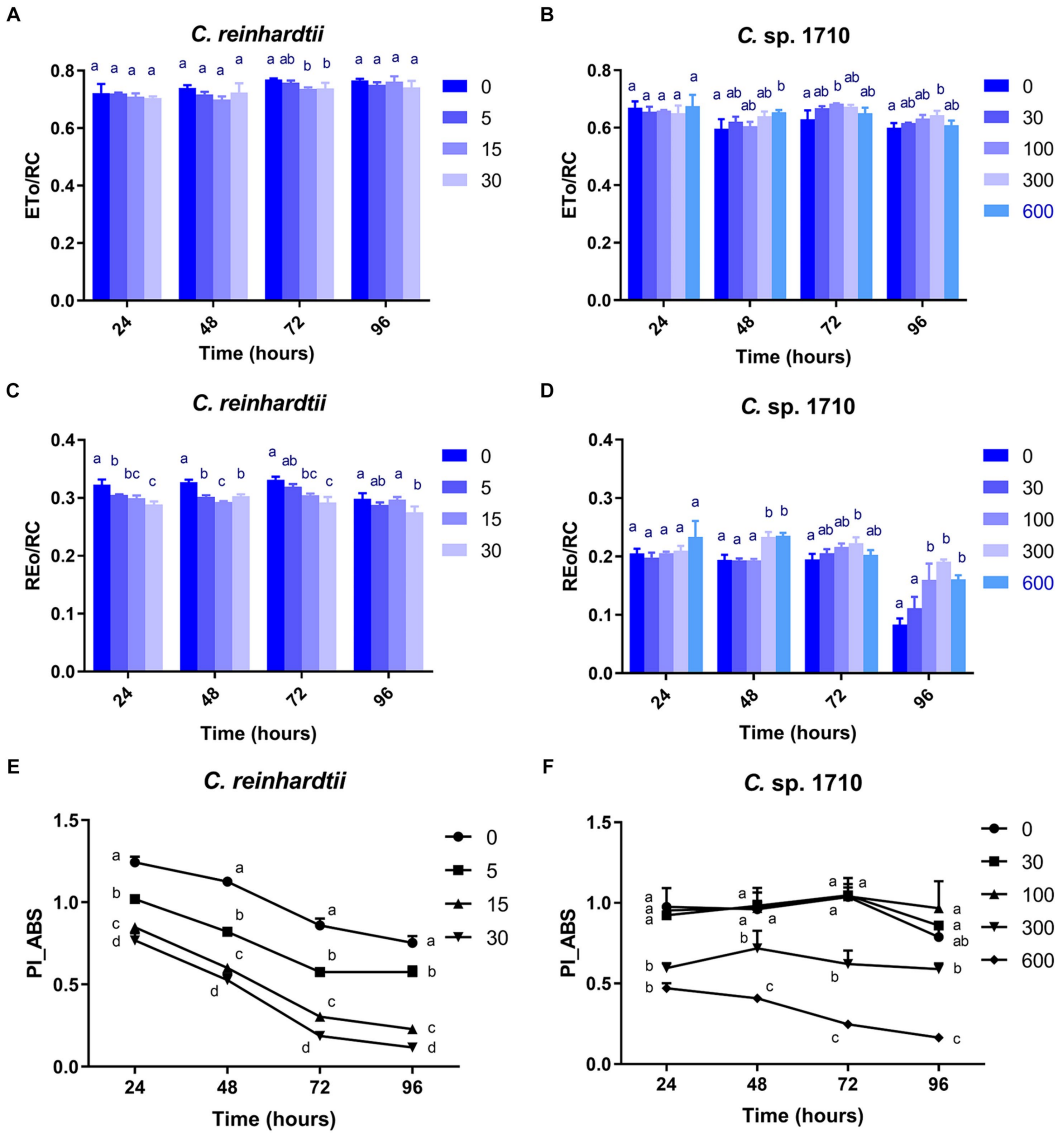
Electron transport flux per reaction center (ETo/RC) **(A,B)**, electron flux reducing end electron acceptors at the PSI acceptor side per reaction center (REo/RC) **(C,D)**, and performance index for energy conservation from photons absorbed by PSII to the reduction of intersystem electron acceptors (PI_ABS) **(E,F)** of *C. reinhardtii* and *Chlamydomonas* sp. 1710 exposed to Zn (mg/L) for 96 h.

The ETo/RC of both *C. reinhardtii* and *Chlamydomonas* sp. 1710 remained constant when exposed to various Zn concentrations (Figures 7A,B), indicating that the electron transport flux per active PSII was unaffected by Zn. In contrast, the result of REo/RC indicated that the electron transfer of *C. reinhardtii* and *Chlamydomonas* sp. 1710 had a different response from Q_A_^−^ to final PSI acceptors per active PSII to Zn. The REo/RC of *C. reinhardtii* exhibited a negative correlation with Zn concentration throughout the entire experimental period (Figure 7C), indicating that the electron flux of *C. reinhardtii* from Q_A_^−^ to the final PSI acceptors was inhibited by Zn (Kalaji et al., 2014). However, the REo/RC of *Chlamydomonas* sp. 1710 tended to increase with Zn concentration lower than 300 mg/L Zn, but decrease when Zn concentration was 600 mg/L (Figure 7D). This result indicated that slight Zn stress had no effect on the electron flux of *Chlamydomonas* sp. 1710 from Q_A_^−^ to the final PSI acceptors, whereas severe Zn stress inhibited this process (Kalaji et al., 2014). This was consistent with previous research in which the PSI and PSII electron transport activities of acidophilic algae increased under suboptimal conditions (Gerloff-Elias et al., 2005), indicating that the PSI of *Chlamydomonas* sp. 1710 remained largely unaffected by 300 mg/L or lower concentrations of Zn (Kalaji et al., 2014). However, the photosynthetic electron transport chain of *C. reinhardtii* could be directly disrupted by heavy metals (Geoffroy et al., 2007; Aksmann et al., 2014). Heavy metals such as aluminum inhibit electron transport from PSII toward PSI of both acidophilic algae and neutrophilic algae, albeit at a lesser extent for acidophiles (Perreault et al., 2010). Therefore, the regulation of photosynthetic electron transfer may explain why *Chlamydomonas* sp. 1710 had a stronger tolerance toward Zn than *C. reinhardtii* (Cardol et al., 2011; Žuna Pfeiffer et al., 2018). A previous study demonstrated that extremophilic *Chlamydomonas* exhibited high cyclic electron flow under salinity stress, along with considerable changes in the expression of photosystem proteins (Kalra et al., 2020). Other mechanisms, such as chlororespiration and state transitions, may also participate in the stress adaptation of acidophilic algae (Gerloff-Elias et al., 2005).

PI_ABS is widely used to assess the response of plants to various types of stress, such as drought, extreme temperature, and nutrient deficiency (Stirbet et al., 2018). As illustrated in Figure 7E, the PI_ABS of *C. reinhardtii* exhibited a strong negative correlation with Zn concentration and decreased from 0.75 to 0.12 when Zn concentration increased from 0 to 30 mg/L, suggesting a direct impairment caused by Zn. In contrast, the PI_ABS of *Chlamydomonas* sp. 1710 was not affected in the same manner. Specifically, 30 and 100 mg/L Zn had no inhibitory effect on the PI_ABS of *Chlamydomonas* sp. 1710 (Figure 7F), indicating that *Chlamydomonas* sp. 1710 had a fairly high level of tolerance to Zn. However, the PI_ABS of *Chlamydomonas* sp. 1710 decreased from 0.79 to 0.16 when Zn concentration increased from 0 to 600 mg/L (Figure 7F). The changing trends of PI_ABS were strongly linked to those of μ, Chl-a content, and quantum yield, indicating that it could be a good parameter reflecting the physiological response of *Chlamydomonas* to Zn stress. Additionally, the result of PI_ABS was consistent with that of NPQ parameters, confirming that *C. reinhardtii* and *Chlamydomonas* sp. 1710 responded to Zn stress in different ways (Spoustová et al., 2013; Singh et al., 2015; Habibi, 2017).

### Correlation between μ, Chl-a content, and chlorophyll fluorescence parameters

Due to the significant differences between various plant and algae species and despite the existence of numerous methods and parameters, a unified and widely applicable measurement and evaluation system for chlorophyll fluorescence has not been developed (Krause and Weis, 1991; Bussotti, 2004; Murchie and Lawson, 2013). Growth rate is the most intuitive and valuable index for evaluating the stress response of *Chlamydomonas* to Zn (Sunda and Huntsman, 1998). However, it is time-consuming and needs to be calculated based on the D-value of at least two time points. The impact of Zn on the growth of algae is regulated by various metabolic and resistant pathways of the algal cell (Esperanza et al., 2015), which cannot be represented by one indicator alone. Nevertheless, examining the relationship between growth rate and other parameters could offer valuable insights for identifying potential indicators of growth rate (Almeida et al., 2021). On the other hand, the level of damage caused by Zn and the underlying mechanisms vary depending on the plant species and the different components of the cells (Vaillant et al., 2005; Tsonev and Cebola Lidon, 2012; Szopiński et al., 2019). For example, Zn primarily affects the photosynthesis of *Triticum durum* by altering the electron transfer from Q_A_ to Q_B_ at the acceptor side of PSII (Paunov et al., 2018). To comprehensively evaluate the effect of Zn on the two *Chlamydomonas* species, PCA was conducted on μ, Chl-a content, and chlorophyll fluorescence parameters.

**Figure 8:**
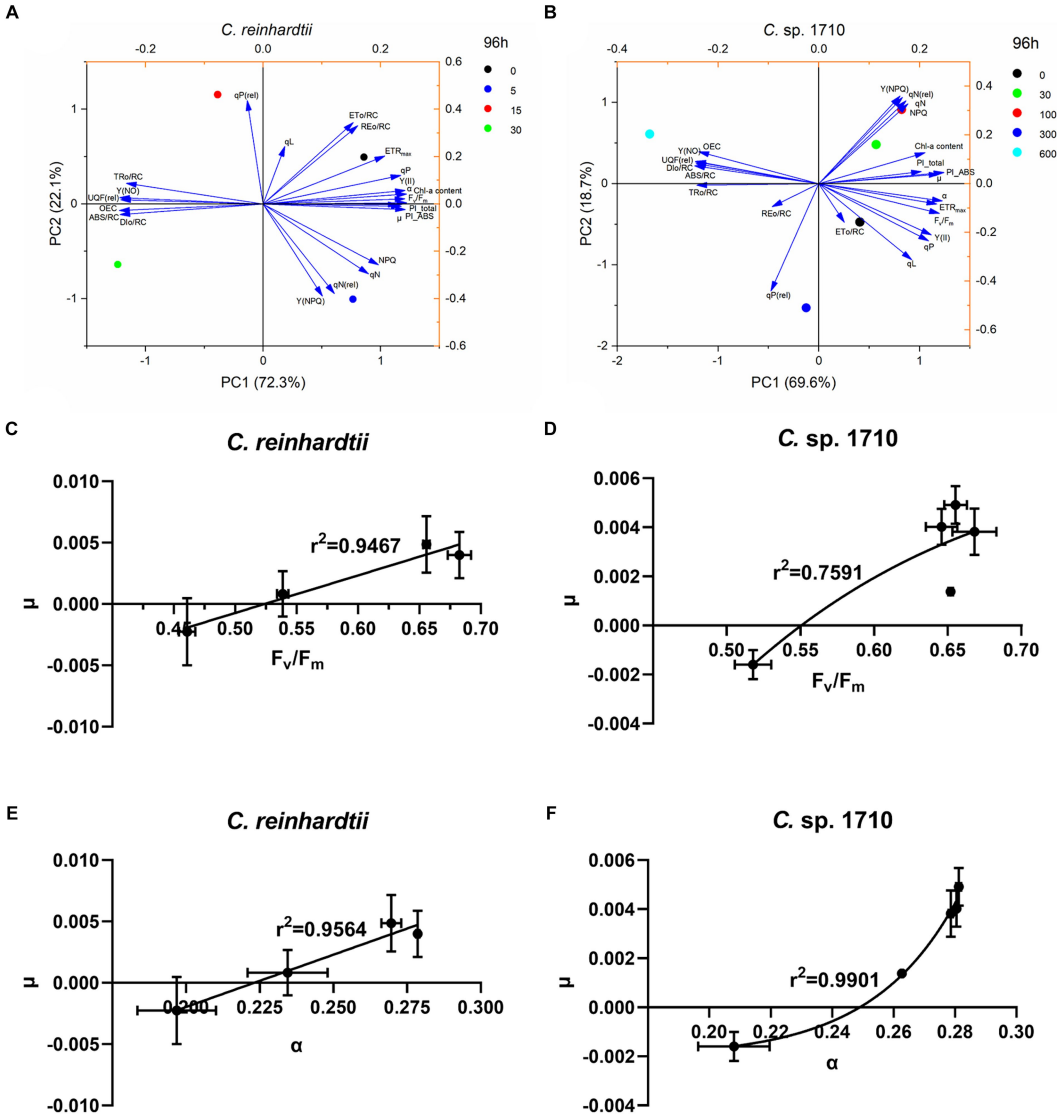
Principal component analysis (PCA) of the specific growth rate (μ), chlorophyll a (Chl-a) content, and chlorophyll fluorescence parameters **(A,B)**, regression models between maximal PSII quantum yield (F_v_/F_m_) and specific growth rate (μ) **(C,D)**, and regression models between light energy utilization efficiency (α) and μ **(E,F)** of *C. reinhardtii* and *Chlamydomonas* sp. 1710 exposed to Zn (mg/L) at 96 h.

PCA was conducted to simplify the response patterns of *Chlamydomonas* upon exposure to Zn for 96 h (Figures 8A,B; Supplementary Figure S3). In the figure, each arrow represents a chlorophyll fluorescence parameter, whereas the circles represent the samples exposed to different Zn concentrations. The direction of the arrows represents the trends of change for each parameter, whereas the arrow length represents the differences among the tested parameters (Machado et al., 2015). The PCA results also appeared to be time-dependent.

For *C. reinhardtii*, PC1 and PC2 explained 94.4% of the total data variance at 96 h. PC1, accounting for 72.3% of the data variance (eigenvalue 16.63), provided a clear distinction among the four Zn concentrations, which were distinctively distributed in four quadrants (Figure 8A). Similar trends were observed for *Chlamydomonas* sp. 1710. At 96 h, PC1 and PC2 explained 88.3% of the total data variance. The five concentrations were approximately distributed in separate quadrants, with 0, 300, and 600 mg/L being distributed in the fourth, third, and second quadrants, respectively, whereas both 30 and 100 mg/L were distributed in the first quadrant (Figure 8B).

Both the control groups (0 mg/L) of *C. reinhardtii* and *Chlamydomonas* sp. 1710 were positively correlated with ETo/RC, qP, Y(II), F_v_/F_m_, ETR_max_, and α (Figures 8A,B). These parameters corresponded to the state of normal growth without stress. For *C. reinhardtii*, 5 mg/L was positively correlated with NPQ, Y(NPQ), qN, and qN(rel), whereas for *Chlamydomonas* sp. 1710, the concentrations were 30 and 100 mg/L (Figure 8A). This indicated that both *Chlamydomonas* strains responded to Zn toxicity via non-photochemical quenching and were able to retain a robust photosynthetic performance (Baracho et al., 2019; Rocha et al., 2021). For *C. reinhardtii*, 15 and 30 mg/L roughly correlated with OEC, Y(NO), UQF(rel), ABS/RC, TRo/RC, and DIo/RC, whereas for *Chlamydomonas* sp. 1710, 300 and 600 mg/L were more closely correlated with the aforementioned parameters (Figure 8B). Consistent with previous studies, these parameters were identified as the main indicators of severe Zn stress (Almeida et al., 2021; Antunović Dunić et al., 2023).

Supplementary Tables S1–S4 summarize the pairwise Pearson correlations of μ, Chl-a content, and all of the examined chlorophyll fluorescence parameters. For *C. reinhardtii*, α (r^2^ = 0.818), F_v_/F_m_ (r^2^ = 0.807) and Y(II) (r^2^ = 0.792) had a highly positive correlation with μ, whereas F_v_/F_m_ (r^2^ = 0.990), Y(II) (r^2^ = 0.987), PI_ABS (r^2^ = 0.977) and α (r^2^ = 0.939) had an extremely significant positive correlation with Chl-a content. In contrast, ABS/RC (r^2^ = −0.836), DIo/RC(r^2^ = −0.831), and OEC (r^2^ = −0.811) had a negative correlation with μ, whereas OEC (r^2^ = −0.979), Y(NO) (r^2^ = −0.977), and ABS/RC (r^2^ = −0.971) had an extremely significant negative correlation with Chl-a content. For *Chlamydomonas* sp. 1710, α (r^2^ = 0.927) and PI_ABS (r^2^ = 0.912) had an extremely significant positive correlation with μ, and PI_ABS (r^2^ = 0.834) and α (r^2^ = 0.823) also had a positive correlation with Chl-a content. In contrast, ABS/RC (r^2^ = −0.894), TRo/RC (r^2^ = −0.928), and DIo/RC (r^2^ = −0.882) had a negative correlation with μ, and ABS/RC (r^2^ = −0.806), TRo/RC (r^2^ = −0.902) and DIo/RC (r^2^ = −0.777) had a negative correlation with Chl-a content. These results were fairly consistent with the findings of previous studies (Almeida et al., 2021; Rocha et al., 2021).

F_v_/F_m_ (Kitajima and Butler, 1975) and Y(II) (Genty et al., 1989) have been widely used to assess the photosynthetic function of plants for decades. In this study, these parameters were highly correlated with the μ of the green alga *C. reinhardtii*, which was consistent with previous research (Li et al., 2013). However, for *Chlamydomonas* sp. 1710, F_v_/F_m_ and Y(II) were not the most correlated parameters with μ, substituted by α and PI_ABS, which were derived from RLC (Ralph and Gademann, 2005) and OJIP (Stirbet and Govindjee, 2011), respectively. These findings confirmed that the patterns of chlorophyll fluorescence parameters were species-specific.

Given that F_v_/F_m_, Y(II), and α had a strong correlation with μ in *C. reinhardtii*, whereas α and PI_ABS were strongly correlated with μ in *Chlamydomonas* sp. 1710, linear and non-linear regression models between these parameters and μ were constructed to determine which model and which chlorophyll fluorescence parameter was a better fit. For the *C. reinhardtii*, fine linear regression models were constructed, with the correlations of F_v_/F_m_ (r^2^ = 0.9564) and α (r^2^ = 0.9467), with μ being better than those of Y(II) (r^2^ = 0.9074) and PI_ABS (r^2^ = 0.826) (Figures 8C–E; Supplementary Figures S4A,C). However, for the *Chlamydomonas* sp. 1710, good linear regression models between these chlorophyll fluorescence parameters and μ could not be constructed, whereas non-linear regression models were more applicable. Furthermore, α (r^2^ = 0.9901) and PI_ABS (r^2^ = 0.9763) clearly exhibited a stronger non-linear regression relationship with μ than F_v_/F_m_ (r^2^ = 0.7591) and Y(II) (r^2^ = 0.7542) (Figure 8F; Supplementary Figures S4B,D). These results indicated that F_v_/F_m_ and α were the optimal parameters reflecting the growth status of *C. reinhardtii*, whereas the growth of *Chlamydomonas* sp. 1710 was more accurately described by α and PI_ABS. Moreover, the growth rate of *Chlamydomonas* sp. 1710 was typically more consistent with the light energy utilization efficiency (r^2^ = 0.9901) than the quantum yield (r^2^ = 0.7591), suggesting that the energy utilization and regulation process of *Chlamydomonas* sp. 1710 was well protected under Zn stress. This aligns with the characteristics exhibited by salt-tolerant algae or cyanobacteria, which possess robust photoprotective mechanisms (Demmig-Adams et al., 2014; Liang et al., 2023). These data are also highly consistent with the result of OJIP parameters that the trapped photons of PSII of *Chlamydomonas* sp. 1710 were clearly lower than those of *C. reinhardtii*.

### Potential implications of the tolerance of *Chlamydomonas* sp. 1710 to Zn manifested by photosynthetic response

Toxic heavy metals are abundant in acidic environments, and thus acidic wastewater endangers natural environments and human health (Simate and Ndlovu, 2014). In this study, Zn was used as a presentative heavy metal to investigate the photosynthetic response of two *Chlamydomonas* species. *Chlamydomonas* sp. *1710* thrives in acidic environments developed unique resistance mechanisms, including antioxidant enzymes and regulation of energy dissipation (Dong et al., 2022; Pluciński et al., 2023; del Carmen Romero-Cruz et al., 2024). The robust tolerance of *Chlamydomonas* sp. 1710 to heavy metals makes it an ideal candidate for addressing heavy metal pollution in acidic environments, such as acid mine drainage. The capability also carries economic benefits, as heavy metals accumulated in algal cells can be recovered, while algal biomass can be utilized for biofuel production (Pavithra et al., 2020; Chakravorty et al., 2023). Understanding the photosynthetic responses of *Chlamydomonas* sp. 1710 to heavy metals helps develop its potential for treating heavy metal pollution under optimized condition, such as adjusting the illumination to achieve appropriate light energy input under heavy metal stress (Ji et al., 2018; Zhao et al., 2023).

## Conclusion

The *Chlamydomonas* sp. 1710 displayed a notably higher resistance to Zn-induced stress compared to the *C. reinhardtii*, as evidenced by a tenfold increase in the IC50 of Zn. The growth and Chl-a content of *C. reinhardtii* were inhibited by 15 mg/L Zn, whereas those of *Chlamydomonas* sp. 1710 was not affected by 100 mg/L Zn. The chlorophyll fluorescence parameters including NPQ, RLC, and OJIP were highly in agreement, indicating that, unlike *C. reinhardtii*, the photosystem of *Chlamydomonas* sp. 1710 possesses outstanding protection mechanisms. Non-photochemical quenching played a crucial role in the energy regulation of both *Chlamydomonas* strains under Zn stress. However, the non-photochemical quenching of *C. reinhardtii* was delayed in the initial 48 h by 15 mg/L Zn, and recovered at 72 h, whereas that of *Chlamydomonas* sp. 1710 remained stable throughout the entire Zn exposure process under 100 mg/L Zn. The quantum yield, light energy utilization efficiency (α), and PI_ABS of *C. reinhardtii* were inhibited by Zn, whereas the passively absorbed and dissipated energy and unregulated energy increased, and these effects could be observed when Zn concentration was only at 5 mg/L. However, although similar effects were exerted on *Chlamydomonas* sp. 1710 by Zn, they were negligible when the Zn concentration was 100 mg/L or lower. The electron flux from Q_A_^−^ to the final PSI acceptor side of *Chlamydomonas* sp. 1710 was more resilient than that of *C. reinhardtii*. The varying trends of ETR_max_ in both the *Chlamydomonas* indicated that 48 h marked the beginning of the adaptation period under Zn stress. The high correlation between the light energy utilization efficiency and the growth rate of *Chlamydomonas* sp. 1710, along with its stable trapped photons of PSII, indicated its energy utilization and regulation process was well protected under Zn stress.

## Data availability statement

The raw data supporting the conclusions of this article will be made available by the authors, without undue reservation.

## Author contributions

DZ: Conceptualization, Data curation, Formal analysis, Investigation, Methodology, Validation, Writing – original draft, Writing – review & editing. YL: Data curation, Investigation, Methodology, Writing – original draft. NY: Data curation, Investigation, Software, Writing – original draft. CH: Funding acquisition, Supervision, Writing – review & editing.

## Appendix

**Table 1:**

Parameter	Definition	Equation	Reference
F_0_	Initial fluorescence	-	Kitajima and Butler (1975)
F_m_	Maximum fluorescence	-	Kitajima and Butler (1975)
F_v_/F_m_	Maximal PSII quantum yield	(F_m_-F_0_)/F_m_	Kitajima and Butler (1975)
OEC	Efficiency of the oxygen-evolving complex	F_0_/(F_m_-F_0_)	Kriedemann et al. (1985)
Y(II)	Effective PSII quantum yield	(F_m_′-F)/F_m_′	Genty et al. (1989)
qP	Coefficient of photochemical quenching	(F_m_′-F)/(F_m_′-F_0_’)*	Schreiber et al. (1986)
NPQ	Non-photochemical quenching	(F_m_-F_m_′)/F_m_′	Bilger and Björkman (1990)
qL	Coefficient of photochemical quenching	(F_m_′-F)/(F_m_′-F_0_’) × F_0_’/F = qP × F_0_’/F*	Kramer et al. (2004)
Y(NPQ)	Quantum yield of regulated energy dissipation	1 – Y(II) – 1/(NPQ + 1 + qL(F_m_/F_0_-1))	Kramer et al. (2004)
Y(NO)	Quantum yield of nonregulated energy dissipation	1/(NPQ + 1 + qL(F_m_/F_0_-1))	Kramer et al. (2004)
qN	Coefficient of non-photochemical quenching	(F_m_-F_m_′)/(F_m_-F_0_’)*	Schreiber et al. (1986)
qP(rel)	Relative photochemical quenching	(F_m_′-F_t_)/(F_m_-F_0_’)	Buschmann (1995)
qN(rel)	Relative non-photochemical quenching	(F_m_-F_m_′)/(F_m_-F_0_’)	Buschmann (1995)
UQF(rel)	Relative unquenched fluorescence	(F_t_-F_0_’)/(F_m_-F_0_’)	Juneau et al. (2005)
V_j_	Relative variable fluorescence at the J-peak of OJIP curve	(F_j_-F_0_)/(F_m_-F_0_)	Strasser et al. (2000)
V_i_	Relative variable fluorescence at the I-peak of OJIP curve	(F_i_-F_0_)/(F_m_-F_0_)	Strasser et al. (2000)
φ_Po_	Maximum quantum yield of primary photochemistry (at *t* = 0)	(F_m_-F_0_)/F_m_ (equal to F_v_/F_m_)	Strasser et al. (2000)
ψ_Eo_	Probability (at *t* = 0) that a trapped exciton moves an electron into the electron transport chain beyond Q_A_^−^	(1-V_j_)	Strasser et al. (2000)
δ_Ro_	Efficiency/Probability with which an electron from the intersystem electron carriers moves to reduce end electron acceptors at the PS I acceptor side	(1-V_i_)/(1-V_j_)	Strasser et al. (2000)
M_0_	Approximated initial slope (ms^−1^) of the fluorescence transient normalized on the maximal variable fluorescence F_v_	4(F_300_-F_0_)/(F_m_-F_0_)	Strasser et al. (2000)
ABS/RC	Absorption flux per reaction center	M_0_(1/V_j_)(1/φ_Po_)	Strasser et al. (2000)
TRo/RC	Trapped energy flux per reaction center	M_0_/V_j_	Strasser et al., 2000
ETo/RC	Electron transport flux per reaction center	(M_0_/V_J_) × ψ_Eo_	Strasser et al. (2000)
DIo/RC	Dissipated energy flux per reaction center	ABS/RC- TRo/RC	Strasser et al. (2000)
REo/RC	Electron flux reducing end electron acceptors at the PSI acceptor side per reaction center	(M_0_/V_J_) × (1-V_i_)	Strasser et al. (2010)
PI_ABS	Performance index for energy conservation from photons absorbed by PSII to the reduction of intersystem electron acceptors	$\frac{RC}{\mathrm{ABS}}\times \frac{\varphi_{Po}}{1-\varphi_{Po}}\times \frac{\psi_{Eo}}{1-\psi_{Eo}}$	Strasser et al. (2010)
PI_total	Performance index for energy conservation from photons absorbed by PSII to the reduction of PSI and acceptors	$\mathrm{PI}:\mathrm{ABS}\times \frac{\delta_{Ro}}{1-\delta_{Ro}}$	Strasser et al. (2010)
α	Light energy utilization efficiency	The initial slope of the ETR-PAR curve	Fu et al. (2012)
ETR	Relative photosynthetic electron transport rate	0.5 x Y(II) x PAR x I_A_	Genty et al. (1989)
ETR_max_	Maximal relative photosynthetic electron transport rate	Maximum of ETR	Genty et al. (1989)

*F_0_’ was calculated by: F_0_’ = F_0_/ (Fv/Fm + F_0_/Fm′) (Oxborough and Baker, 1997).
