# Supplementary figures and images for: Photosynthetic response of Chlamydomonas reinhardtii and Chlamydomonas sp. 1710 to zinc toxicity
Source: Front Microbiol. 2024 Apr 8;15:1383360. doi: 10.3389/fmicb.2024.1383360 (PMC11033396; doi:10.3389/fmicb.2024.1383360)

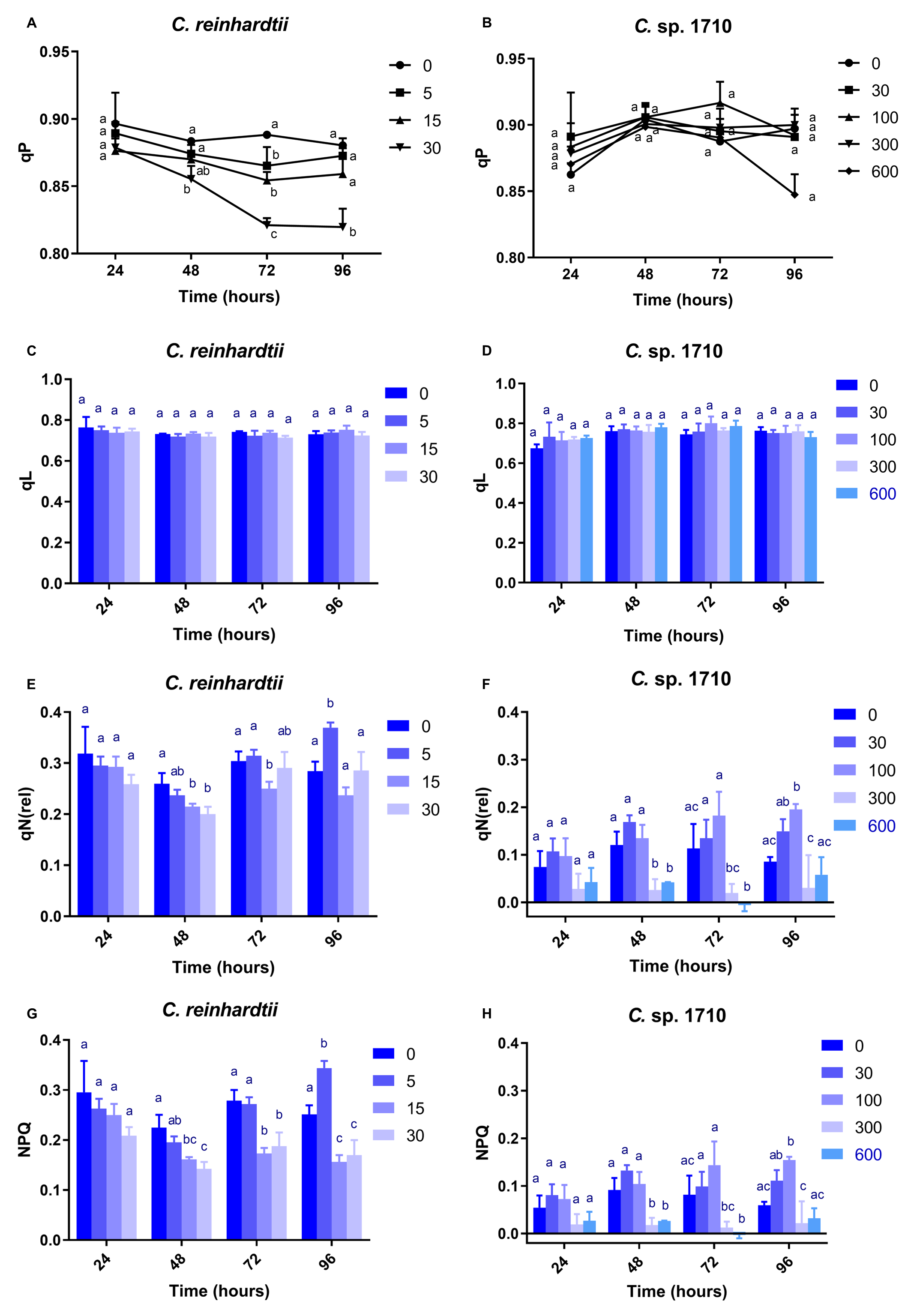

Supplement: Supplementary file 1 [file Image_1.TIF]

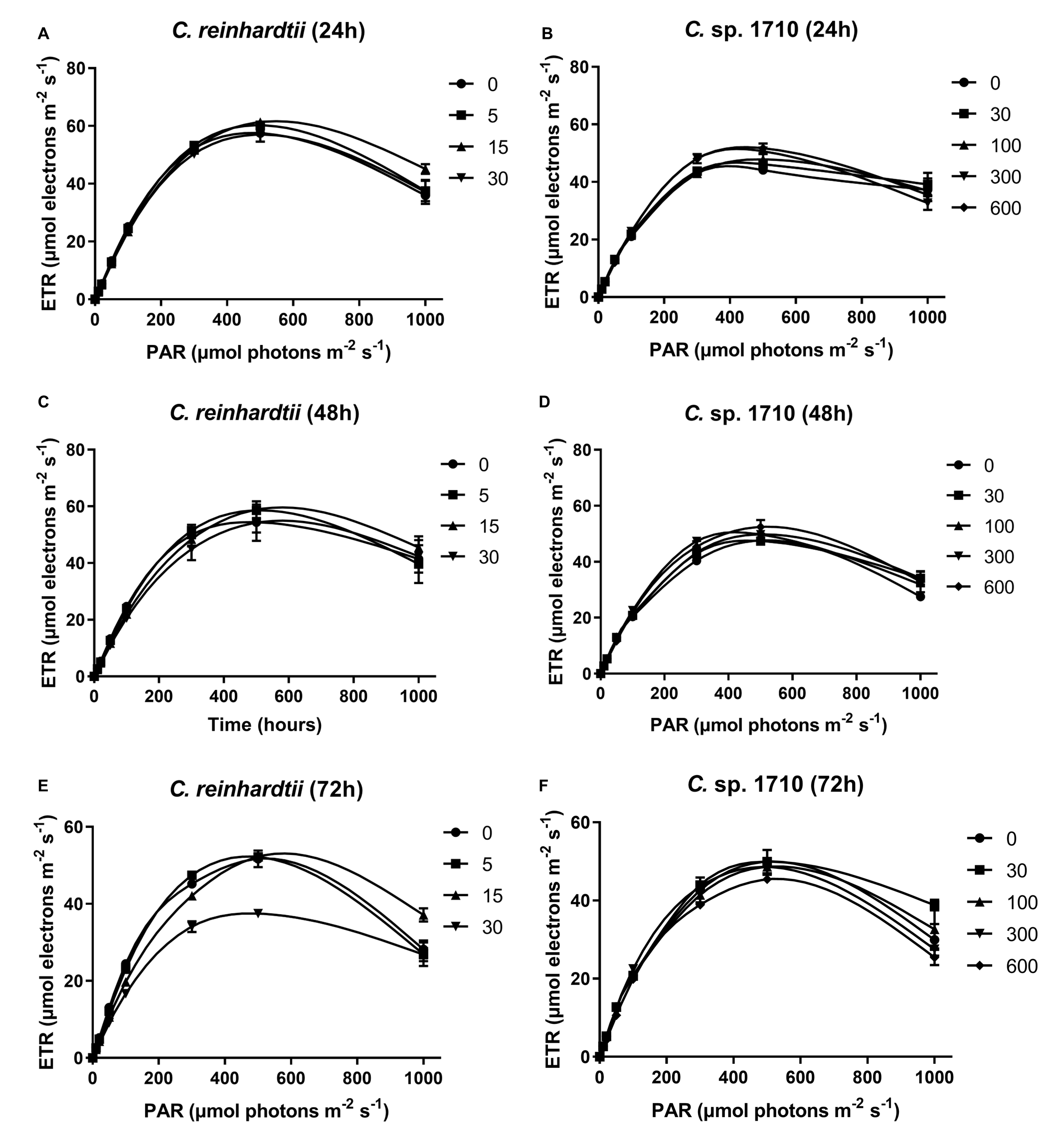

Supplement: Supplementary file 2 [file Image_2.TIF]

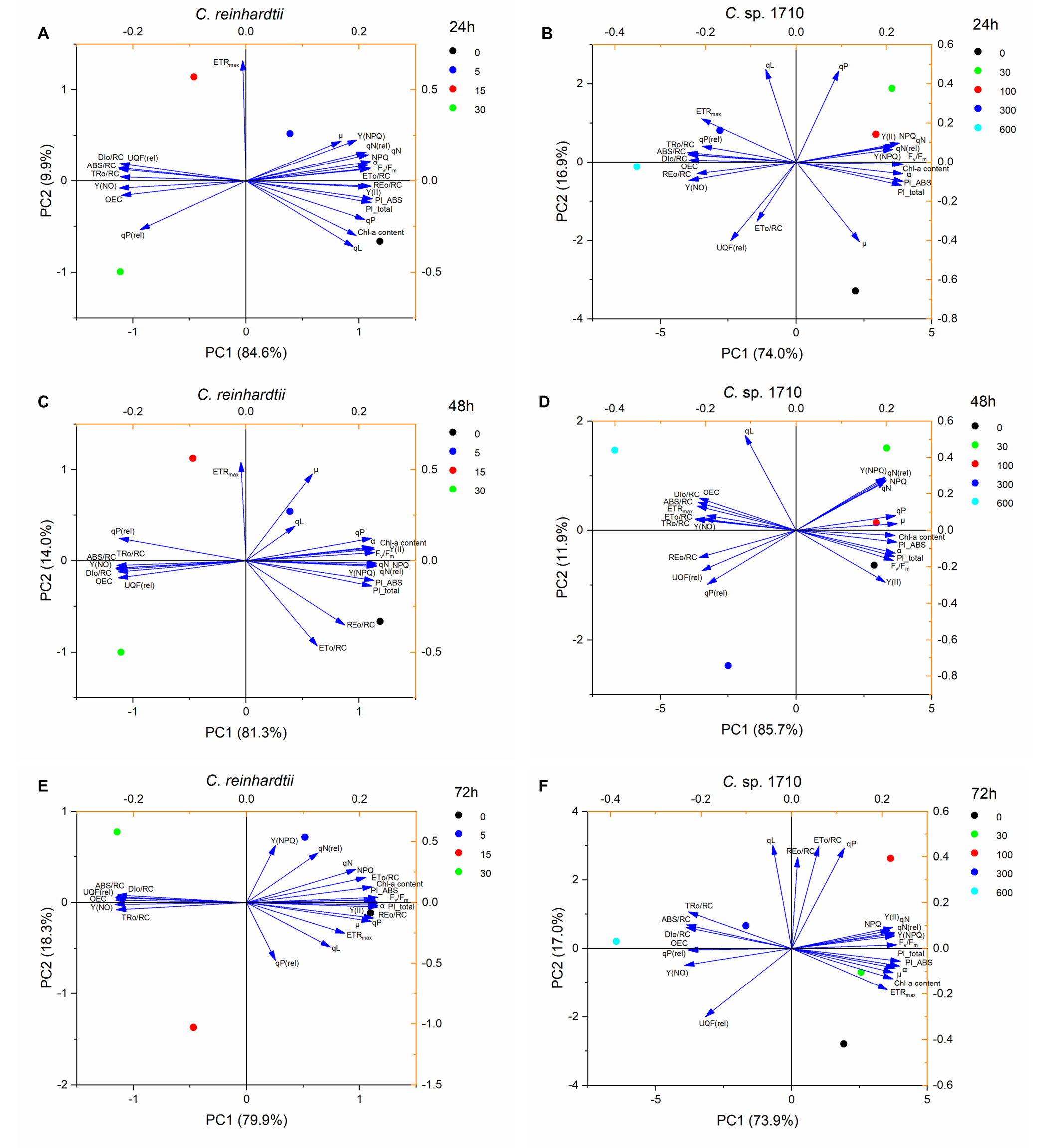

Supplement: Supplementary file 3 [file Image_3.TIF]

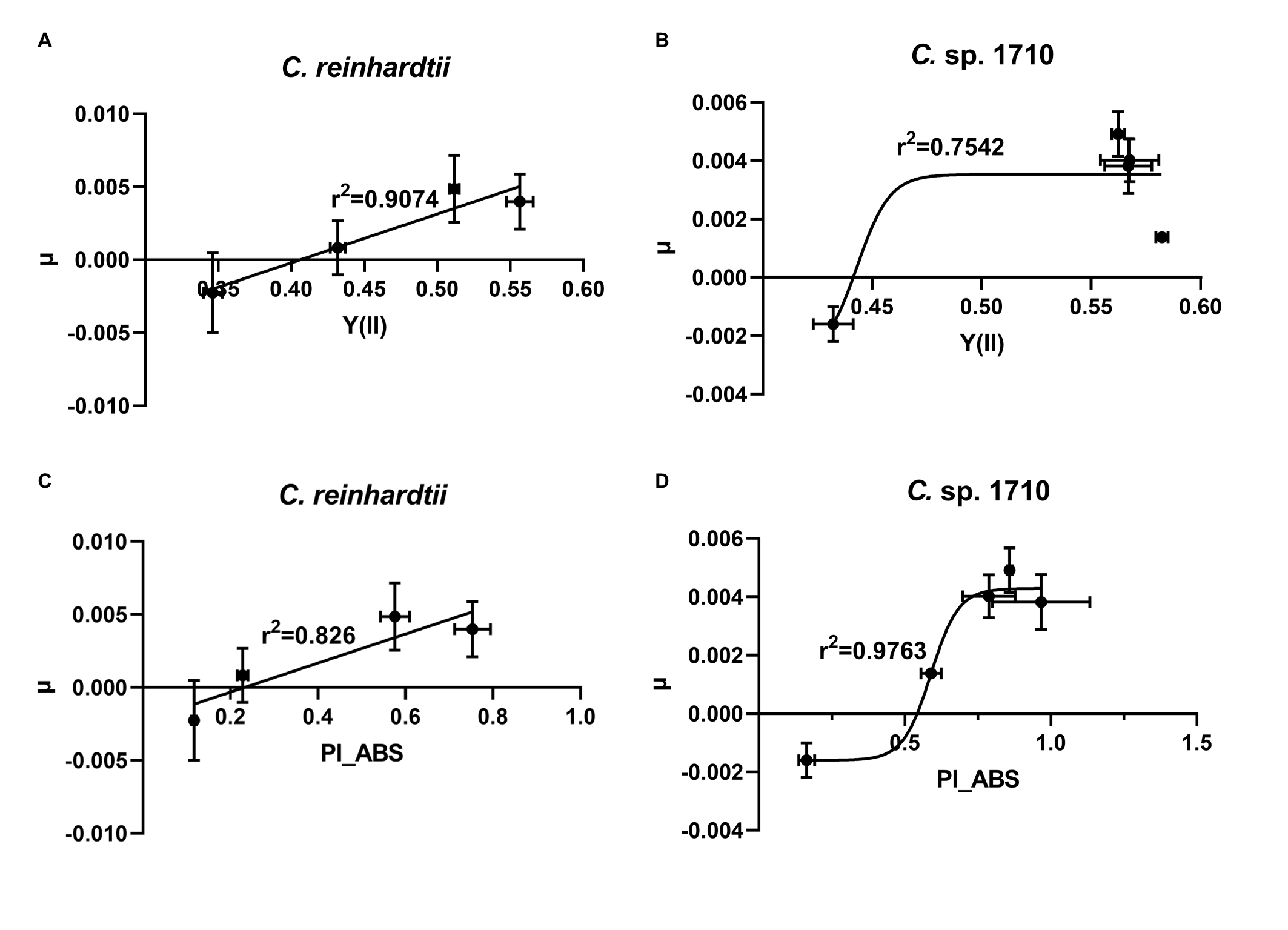

Supplement: Supplementary file 4 [file Image_4.TIF]
