# Supplementary material for: Photosynthetic response of Chlamydomonas reinhardtii and Chlamydomonas sp. 1710 to zinc toxicity
Source: Front Microbiol. 2024 Apr 8;15:1383360. doi: 10.3389/fmicb.2024.1383360 (PMC11033396; doi:10.3389/fmicb.2024.1383360)
Supplement: Supplementary file 5 [file Table_1.DOCX]

Figure S1 Coefficient of photochemical quenching (qP) (A,B), coefficient of photochemical quenching (qL) (C,D), relative non-photochemical quenching (qN(rel)) (E,F), and non-photochemical quenching (NPQ) (G,H) of *C. reinhardtii* and *Chlamydomonas* sp. 1710 exposed to Zn (mg/L) in 96 h.

Figure S2 Rapid light curves of *C. reinhardtii* (A) and *Chlamydomonas* sp. 1710 (B) exposed to Zn (mg/L) at 24h, 48h, and 72 h.

Figure S3 Principal component analysis (PCA) of *C. reinhardtii* (A) and *Chlamydomonas* sp. 1710 (B) exposed to Zn (mg/L) at 24h, 48h, and 72 h.

Figure S4 Linear and non-linear regression models between effective PSII quantum yield and specific growth rate (μ) (A,B), and between performance index for energy conservation from photons absorbed by PSII to the reduction of intersystem electron acceptors (PI_ABS) and μ (C,D) of *C. reinhardtii* and *Chlamydomonas* sp. 1710 exposed to Zn at 96 h.
